# Benign orbital angiomatous tumors with intracranial extension

**DOI:** 10.1186/s40001-015-0157-x

**Published:** 2015-08-12

**Authors:** Konrad R Koch, Mario Matthaei, Stefan J Grau, Tobias Blau, Edwin Bölke, Ole Schlichting, Claus Cursiefen, Ludwig M Heindl

**Affiliations:** Department of Ophthalmology, University of Cologne, Cologne, Germany; Department of Neurosurgery, University of Cologne, Cologne, Germany; Department of Neuropathology, University of Cologne, Cologne, Germany; Department of Radiology and Radiooncology, University of Duesseldorf, Duesseldorf, Germany; Department of Radiation Oncology, University of Duesseldorf, Duesseldorf, Germany

**Keywords:** Bone erosion, Orbital tumor, Intracranial extension, Malignancy, Hemangioma

## Abstract

Orbital neoplasms with associated bone erosions and intracranial extension are generally considered suspicious for malignancies. Here, we describe the clinical and radiological findings, as well as the surgical management of two extraordinary cases, in which such bony perforations with subsequent intracranial tumor growth resulted from benign angiomatous orbital neoplasms. Two female patients, 69 years old (case 1) and 51 years old (case 2), had both developed visual symptoms (visual field restrictions and/or visual acuity loss) over several months. Computed tomography revealed an orbital tumor of the anterosuperior orbit with painless swelling of the medial upper eyelid of the right eye in case 1, and a posterior intraconal tumor close to the orbital apex of the left eye in case 2, respectively. In both cases, the tumor was associated with a perforation of the orbital roof connecting the orbit with the anterior cranial fossa. An interdisciplinary ophthalmologic and neurosurgical approach allowed for complete tumor removal, in both patients with no signs for local recurrence during the subsequent follow-up of 15 and 18 months, respectively, as well as for a satisfactory visual rehabilitation.

## Background

Orbital tumors although relatively rare with an estimated incidence of around 3 cases in one million people comprise a variety of benign and malignant neoplastic entities [1, 2]. Given that the orbit is encircled laterally and posteriorly by bony walls, these tumors as space-consuming lesions become most often clinically evident by an axial protrusion of the ipsilateral eye (proptosis) [3–5]. Depending on the primary tumor localization within or outside the extraocular muscle cone, proptosis can be associated with a significant downward or lateral displacement of the eye globe. With growing tumor diameters compression of the optic nerve can result in irreversible functional deterioration including visual acuity loss and visual field defects. Depending on the degree of proptosis reduced ocular surface lubrication and incomplete lid closure may cause additional corneal disease. Impaired motility of the ipsilateral extraocular muscles with subsequent diplopia may equally appear.

In order to choose an adequate diagnostic as well as therapeutic approach, not only tumor dimensions and localization have to be considered but also and as importantly the benign versus malignant character of the lesion. While benign tumors may be observed and assessed conservatively as long as the (bin)ocular function remains unaffected [6], malignant entities require an immediate and more radical treatment, which apart from surgical tumor removal by means of an orbitotomy may enclose adjuvant chemo- or radiotherapeutical measures.

In turn, incisional and excisional orbital surgery is demanding, often requiring a multidisciplinary approach of orbitoplastic, neurosurgical, and maxillofacial surgical specialists, and is associated by itself with a significant risk for the ocular function [7, 8]. The surgical indication should therefore be weighed carefully always based on clinical, echographical, and radiological findings.

Commonly, tumor derived perforations of the bony orbit are among those radiological features raising suspicion for a malignancy, thus also impacting therapeutical decisions. However, very rarely bony erosions and intracranial tumor extension may derive from benign orbital tumors as well, which we demonstrate with this series of two patients, who presented between 09/2013 and 03/2014 to our oculoplastic and ophthalmo-oncological service at the Department of Ophthalmology, University of Cologne, Cologne, Germany, with anterior (case 1) and posterior (case 2) orbital neoplasms of primarily uncertain behavior.

## Case presentation

### Case 1

**Fig. 1:**
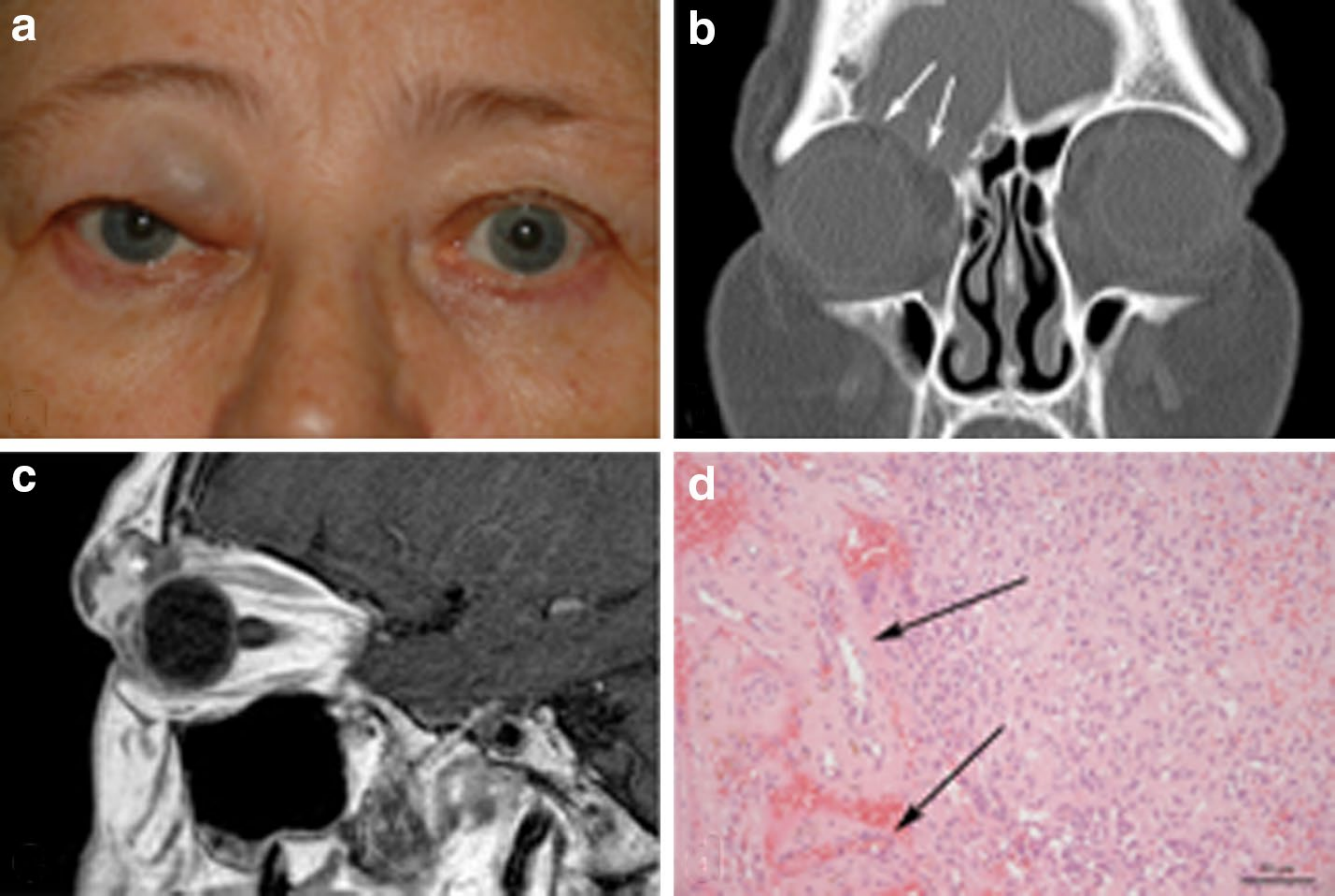
Anterior orbital mass in the right eye of a 69-year-old patient (case 1). **a** Livid swelling of the medial upper lid causing visual field restriction. **b** Computed tomography revealing a large-scale defect of the orbital roof (*white arrows*). **c** T1-weighted contrast-enhanced magnetic resonance tomography showing a round lesion with irregular hyperintensity (*asterisk*). **d** Histopathological section showing a arteriosus malformation as well as components of a capillary hemangioma (*arrows*, H&E staining, original magnification ×200).

A 69-year-old female patient was referred to our hospital in 09/2013 with a tumor of the anterosuperior orbit accompanied by a pea-sized livid painless swelling of the medial upper eyelid of the right eye. Based on the history and portrait photographs, a significant increase in tumor size could be assessed over an 18-month-period. According to the anterior localization in the orbital entrance Hertel exophthalmometry revealed no protrusion of the ipsilateral eye globe. Functionally, the tumor did not affect best-corrected visual acuity (BCVA, 20/20 right eye) or ocular motility. However, swelling of the upper eyelid (Fig. 1a) resulted in a restriction of the superior visual field. Radiologically, computed tomography (CT) images revealed a well-circumscribed tumor with a maximum diameter of 17.0 mm and large-scale loss of the anterior orbital roof connecting the orbit with the anterior cranial fossa (Fig. 1b). In T1-weighted magnetic resonance imaging (MRT) the tumor signal was isointense compared to the extraocular muscles before contrast application and showed an inhomogeneous contrast medium uptake (Fig. 1c). Due to documented tumor growth, to the perimetric symptoms, and to the large-scale bone erosion of the orbital roof connecting the orbit with the anterior cranial fossa, the patient underwent excisional surgery in 01/2014. Since the tumor was pediculated through the bony perforation widely stretching into the frontobasal dura, complete excision by means of an anterior transcutaneous orbitotomy alone was not possible, but was achieved with an additional small orbital craniotomy with focal dural resection and cauterization. Histopathologically, the tumor was composed of partly small capillary vessels and partly larger vascular malformations, areas of fresh hemorrhages and hemosiderin deposits, embedded in mature adipose as well as fibrotic connective tissue. Immunohistochemical staining was positive for blood endothelial markers CD31 and CD34, while being negative for lymphatic endothelial marker LYVE-1, and for melanocytic marker S-100. Staining of proliferation marker Ki-67 revealed only singular immune-activated leucocytes. Accordingly, the orbital tumor was diagnosed as capillary hemangioma associated with an arteriovenous malformation. After resorption of the postsurgical lid edema, no residual visual field defects were detectable (OD). During the subsequent 18-months-follow-up, repeated clinical and echographic monitoring and MRT imaging revealed no signs for an orbital or frontobasal tumor recurrence.

### Case 2

**Fig. 2:**
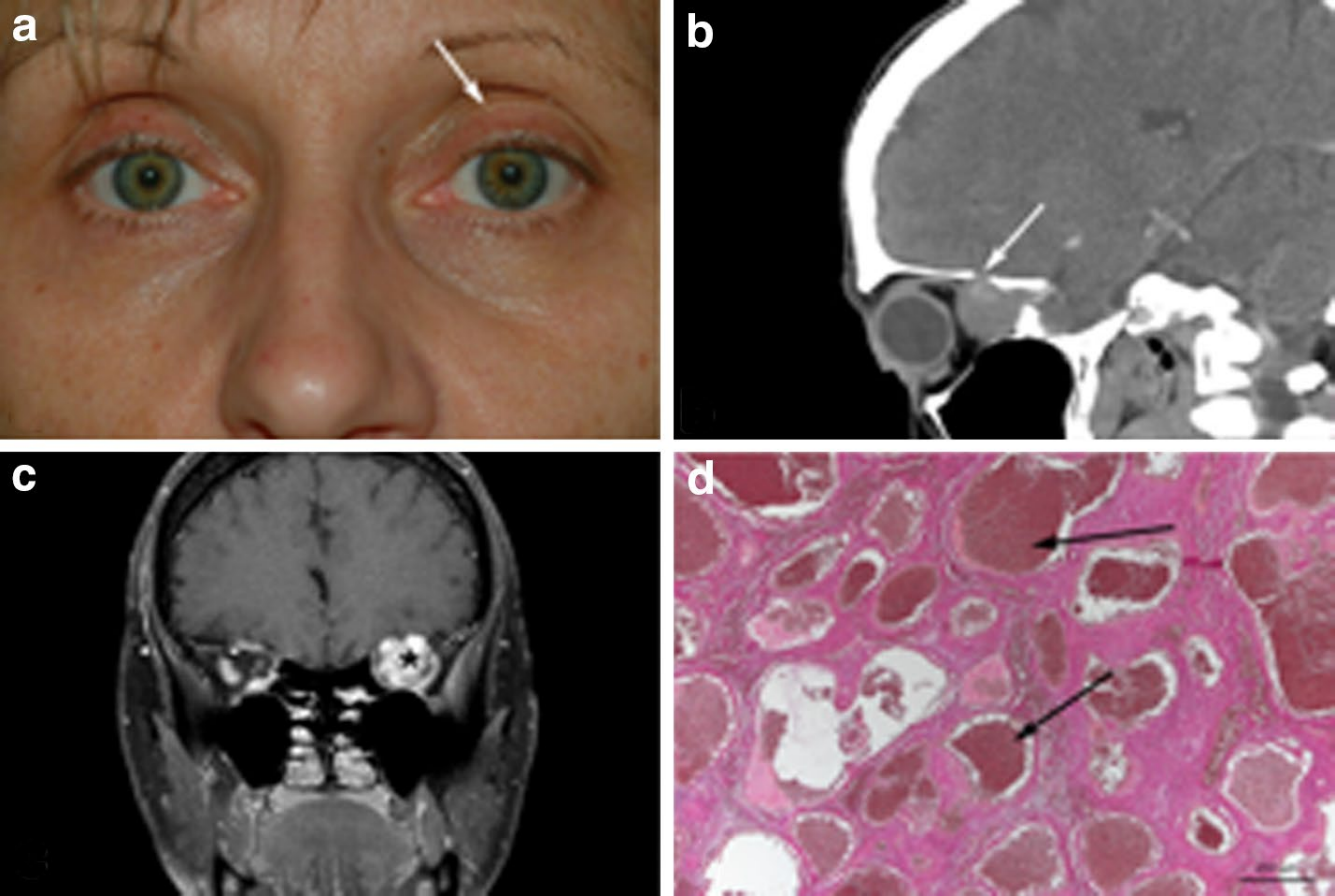
Posterior intraconal tumor in the left orbit of a 51-year-old female patient (case 2). **a** Forward shift of the upper lid skin crease as sign of mild proptosis (*arrow*). **b** Bone perforation connecting the orbit with the anterior cranial fossa (*arrow*, computed tomography). **c** Irregular contrast enhancement of the well-circumscribed round tumor after on T1-weighted magnetic resonance tomography (*asterisk*) impairing visual acuity. **d** Histopathologic evaluation showing large cavernous vessels (*arrows*), confirming the diagnosis of a hemangioma (Elastica van Gieson staining, original magnification ×50).

A 51-year-old female patient presented to our clinic in 03/2014 reporting a progressive visual impairment in her left eye over 6 months. BCVA was reduced to 20/40 and 30° perimetry showed a concentric visual field restriction. Hertel exophthalmometry showed a slight 2 mm proptosis of the left eye (Fig. 2a). Ocular motility was unaffected. CT imaging revealed a round, well-circumscribed posterior intraconal tumor close to the orbital apex, with a maximum diameter of 16.3 mm surrounding the optic nerve with an adjacent perforation of the superior orbital bony wall towards the anterior cranial fossa (Fig. 2b). In MRT scans, similar to case 1 the tumor was isointense to the extraocular muscles (hypointense to the orbital fat) before contrast enhancement, while showing irregular hyperintensity following contrast medium uptake (Fig. 2c). In view of the visual symptoms attributable to compressive optic neuropathy surgical intervention was mandatory. Due to the tumor localization in the orbital apex, a transcranial orbitotomy approach with pterional craniotomy was chosen, which allowed for complete excision of the intraconal tumor, which did not adhere to the optic nerve. Histopathological evaluation showed a hemangioma of a mixed capillary and cavernous type (Fig. 2d), thereby confirming the macroscopic assumption during surgery. In the postsurgical course, proptosis of the left eye resolved completely. Visual field defects were slightly regressing and visual acuity increased to 20/30. During the 15-month follow-up, no sign of tumor recurrence was clinically and radiologically detectable.

Vascular tumors, particularly cavernous hemangiomas in adults and capillary hemangiomas in infants, belong to the most frequent primary benign orbital neoplasms [9]. As the present cases typically demonstrate, these tumors can be located in the anterior (orbital entrance) or in the posterior orbit (orbital cone and apex), in the intraconal and less often in the extraconal space [9]. Also with regard to their well-demarcated and rather spherical shape, the tumors of this series can be considered representative [10]. However, vascular as well as other benign tumors are by definition incapable of invading neighboring tissues, thus primarily raising the suspicion for malignancy when bony erosions adjacent to an orbital neoplasm become radiologically apparent. Indeed, perforations of the bony orbital roof resulting in a patent connection between orbit and cranial cavity, which are caused by the angiomatous benign tumors described here, are highly exceptional. An extensive literature search revealed only three previously published single cases of bone erosions caused by primary orbital hemangiomas [11–13], among which only one showed a perforation of the orbital roof [12]. The present cases demonstrate that such orbital roof perforations can originate from benign angiomatous tumors of both, the orbital entrance and the orbital apex. In the single case described by Yan et al., the orbital roof defect was associated with sound adhesion of the hemangioma to the surrounding tissues thus aggravating the operation [12]. This observation corresponds to the surgical findings in our case 1, where strong adhesion with the frontobasal dura required a focal durectomy. Such adhesion complicates and prolongs surgery significantly apart from the potential need to reconstruct bony defects using implants such as resorbable polydioxanon foils.

Among the mechanisms, which might contribute to bone destruction adjacent to benign orbital tumors, a pressure-induced atrophy might as well come into question as a pro-osteoclastic growth factor milieu derived from tumor surrounding inflammatory cells [14].

## Conclusions

Bone erosions related to tumoral orbital lesions, even though remaining suspect for malignant neoplastic entities may be the result of angiomatous benign lesions. Our cases and the few previously published ones underline that the assumed diagnosis, the differentiation between benign and malignant entities, and the subsequent urgency for surgical interventions should be based on as many clinical and radiological criteria as available, rather than on isolated findings that might lack sufficient sensitivity and specificity [15].

If surgery of orbital neoplasms with bone perforations and intracranial extension is intended, a more complicated and prolonged surgical course should be anticipated, since stronger tissue adhesions might be found. Since little invasive extracranial approaches usually performed by orbital and ophthalmic plastic surgeons are unsuitable to make such tumors accessible and resectable as a whole, transcranial interdisciplinary approaches are mandatory, allowing for complete tumor removal as well as for rehabilitation of visual functions.

## Consent

Written informed consent was obtained from the patients for publication of this case report and any accompanying images. A copy of the written consent is available for review by the Editor-in-Chief of this journal.
